# Supplementary material for: Different Risk Factors for Erosive Tooth Wear in Rural and Urban Nepal: A National Study
Source: Int J Environ Res Public Health. 2021 Jul 22;18(15):7766. doi: 10.3390/ijerph18157766 (PMC8345451; doi:10.3390/ijerph18157766)
Supplement: Supplementary file 1 [file ijerph-18-07766-s001.zip › ijerph-1284671-supplementary.pdf]

Table S1. Association between erosive tooth wear (sum score 0–2 / ≥3) and explanatory variables (gender, location, school types, ethnic group, fizzy drinks consumption, fruits consumption, toothbrushing frequency, use of toothpaste, use of charcoal, use of miswak, body mass index, waist-hip ratio, and waist-height ratio) using logistic regression models for children (5–6-year-olds) and adolescents (12- and 15-year-olds).

Explanatory variables	Model 1 UOR (95% CI)	Model 2 UOR (95% CI)
Gender		
Boys	0.94 (0.58–1.53)	1.07 (0.74–1.55)
Girls	1	1
Location		
Urban	0.64 (0.39–1.04)	0.41 (0.28–0.60) **
Rural	1	1
School types		
Private	0.76 (0.46–1.23)	0.37 (0.23–0.59) **
Public	1	1
Ethnic group		
Brahman/Chhetri	1.65 (0.93–2.95)	1.38 (0.90–2.11)
Tarai Madeshi & Muslim	1.65 (0.53–5.13)	1.33 (0.64–2.77)
Dalit	2.24 (0.96–5.20)	1.40 (0.71–2.80)
Janajati & Newar	1	1
Fizzy drinks consumption		
Frequent	0.79 (0.41–1.53)	1.08 (0.69–1.70)
Never & seldom	1	1
Fruits consumption		
Frequent	1.34 (0.72–2.46)	0.97 (0.67–1.41)
Never & seldom	1	1
Toothbrushing frequency		
Once daily or less	0.32 (0.11–0.93) *	1.33 (0.84–2.10)
Twice daily or more	1	1
Use of toothpaste		
Yes	0.52 (0.15–1.85)	-
No	1	-
Use of charcoal		
Yes	3.35 (0.66–17.09)	2.51 (1.44–4.37) *
No	1	1
Use of miswak		
Yes	1.65 (0.29–9.23)	1.24 (0.69–2.21)
No	1	1
Body mass index		
Low	1.58 (0.70–3.56)	0.98 (0.57–1.68)
High	0.96 (0.41–2.23)	0.82 (0.39–1.71)
Normal	1	1
Waist-Hip ratio		
Obese	1.92 (1.02–2.62) *	1.30 (0.80–2.13)
Normal	1	1
Waist-Height ratio		
Obese	1.11 (0.61–2.01)	1.12 (0.45–2.76)
Normal	1	1

*$p < 0.05$; **$p < 0.001$; UOR: unadjusted odds ratio; 95 % CI: 95% confidence interval. Model 1 was performed for children aged 5–6-years; Model 2 was performed for adolescents aged 12- and 15-year-olds.
